# Ultrasound-Guided Thoracic Paravertebral, Erector Spinae Plane and Quadratus Lumborum Blocks During Emergency Laparotomy in Dogs: A Multicenter Retrospective Study

**DOI:** 10.3390/vetsci13090950

**Published:** 2026-09-12

**Authors:** Andrea Paolini, Matteo Serpieri, Amanda Bianchi, Giuseppe Bonaffini, Pedro Figueirinhas, Mitzy Mauthe von Degerfeld, Claudia Ristori, Roberto Tamburro

**Affiliations:** 1 Department of Veterinary Sciences, University of Teramo, Strada Provinciale 18, Piano D’Accio, 64100 Teramo, Italy; apaolini@unite.it (A.P.); abianchi@unite.it (A.B.); claudia_ristori@yahoo.it (C.R.); rtamburro@unite.it (R.T.); 2 Department of Veterinary Sciences, University of Turin, Largo Braccini 2, 10095 Grugliasco, Italy; giuseppe.bonaffini@unito.it (G.B.); mitzy.mauthe@unito.it (M.M.v.D.); 3 Department of Animal Pathology, University of Las Palmas de Gran Canaria, Trasmontana S/N, 35416 Arucas, Spain

**Keywords:** locoregional anesthesia, opioid-sparing, thoracic paravertebral block, erector spinae plane block, quadratus lumborum block, hemodynamic stability

## Abstract

Emergency laparotomy is frequently performed in dogs with cardiovascular compromise, making perioperative anesthesia and nociceptive management particularly challenging. Although ultrasound-guided regional anesthesia techniques have demonstrated opioid-sparing effects during elective procedures, evidence supporting their use in emergency surgery remains limited. Medical records of 61 client-owned dogs were reviewed, and 41 met the inclusion criteria. Dogs were retrospectively allocated to one of four groups according to the analgesic technique received: thoracic paravertebral (TPV, n = 12), erector spinae plane (ESP, n = 11), quadratus lumborum (QL, n = 7), or no regional anesthesia (comparison group, n = 11). All regional blocks were performed using ropivacaine. Total intraoperative fentanyl consumption differed significantly among groups (*p* < 0.0001), with all regional anesthesia groups requiring less fentanyl than dogs managed without a regional block. Block execution time also differed significantly, with the ESP block requiring the shortest execution time. No significant differences were observed in block-related complications. Intraoperative hypotension did not differ significantly among the regional anesthesia groups; however, significant differences were observed between the control group and both the TPV and ESP groups. Ultrasound-guided TPV, ESP, and QL blocks were associated with lower intraoperative rescue fentanyl requirements than systemic analgesia alone, while block-related technical complications were uncommon. These techniques may represent useful components of multimodal anesthesia for selected dogs undergoing emergency laparotomy, providing opioid-sparing effects without increasing the incidence of intraoperative hypotension compared with systemic opioid analgesia alone.

## Simple Summary

Emergency laparotomy in dogs is associated with nociceptive stimulation and is frequently performed in animals with cardiovascular compromise. In these cases, minimizing systemic opioid administration while maintaining effective analgesia may be clinically advantageous. This retrospective multicenter observational study evaluated three ultrasound-guided regional anesthesia techniques performed using ropivacaine—thoracic paravertebral, erector spinae plane, and quadratus lumborum blocks—and compared them with systemic analgesia alone. Dogs receiving regional anesthesia required less intraoperative rescue fentanyl than those managed without a regional block, while block-related technical complications were uncommon. These findings support the use of ultrasound-guided regional anesthesia as part of a multimodal analgesic strategy for selected dogs undergoing emergency laparotomy.

## 1. Introduction

Emergency laparotomy is commonly required in dogs presenting with life-threatening abdominal conditions associated with varying degrees of hypovolemia, hemorrhage, systemic inflammation and cardiovascular compromise, making anesthetic management particularly challenging [1,2]. Although adequate resuscitation should precede surgery whenever possible, definitive surgical treatment may not be safely delayed until all physiological abnormalities have been corrected. Consequently, perioperative analgesia should provide effective nociceptive control while limiting additional cardiovascular depression. In addition, attenuation of the anesthetic-surgical stress response may be particularly relevant in emergency patients with pre-existing systemic and cardiovascular compromise. Surgical trauma activates neuroendocrine, metabolic, inflammatory, and hemodynamic responses that, when excessive or prolonged, may adversely affect perioperative homeostasis and recovery. Regional anesthetic techniques using local anesthetics may contribute to attenuating this response by reducing afferent nociceptive transmission and the consequent neuroendocrine activation, while decreasing the requirements for systemic anesthetic and analgesic agents [3,4]. Regional anesthesia is therefore increasingly incorporated into multimodal veterinary anaesthetic protocols because it can provide procedure-specific analgesia and reduce the requirements for systemically administered analgesic and anaesthetic agents [5,6]. Ultrasound-guided thoracic paravertebral (TPV), erector spinae plane (ESP) and quadratus lumborum (QL) blocks have been investigated as regional analgesic techniques for thoracic and abdominal procedures in dogs. ESP and QL blocks are interfascial plane techniques, whereas TPV block involves the deposition of local anaesthetic within or adjacent to the thoracic paravertebral space. These techniques primarily target somatic afferent pathways; depending on the approach and extent of injectate spread, TPV and some ESP or QL approaches may also involve structures associated with visceral nociceptive transmission [6,7,8,9,10].

Anatomical and clinical studies have reported potentially useful injectate distribution, reduced perioperative opioid requirements and acceptable analgesia in selected elective procedures, although the amount and quality of evidence vary considerably among techniques [7,8,9,10,11].

Their clinical performance in dogs undergoing emergency abdominal surgery remains poorly characterized. Compared with animals undergoing elective procedures, dogs requiring emergency laparotomy frequently present with hemodynamic instability, systemic inflammation, hemorrhage or other physiological derangements that may affect both the choice and the clinical consequences of regional analgesic techniques [1,2]. Although isolated clinical reports are available, comparative clinical evidence evaluating these regional techniques in emergency abdominal surgery is currently lacking.

Therefore, the aim of this multicenter retrospective study was to compare perioperative outcomes among dogs receiving TPV, ESP or QL blocks and dogs receiving systemic analgesia without a regional block. We hypothesized that the use of a regional block would be associated with lower intraoperative opioid requirements without increasing the incidence of block-related complications and, as a secondary hypothesis, with a lower incidence of intraoperative hypotension compared with systemic analgesia alone.

## 2. Materials and Methods

### 2.1. Study Design and Ethical Considerations

This multicenter retrospective observational study evaluated perioperative outcomes associated with three ultrasound-guided regional anesthesia techniques in dogs undergoing emergency laparotomy. Medical records of client-owned dogs treated between January 2023 and March 2026 were reviewed at two referral hospitals: the Veterinary Teaching Hospital “Ruggero Bortolami”, University of Teramo (Italy), and the Veterinary Teaching Hospital of the University of Las Palmas de Gran Canaria (Spain). All anesthetic and surgical procedures were performed as part of routine emergency clinical practice.

Regional anesthesia and anesthetic management were performed by anesthetists experienced in ultrasound-guided regional techniques (A.P., C.R., P.F.). The choice of locoregional technique reflected the attending anesthetist’s clinical judgment, institutional practice and individual patient characteristics rather than random treatment allocation. Written owner consent for anesthesia and surgery was obtained before all procedures. According to institutional policies, formal ethical approval was not required because all procedures were performed exclusively for clinical purposes and no intervention was undertaken for research purposes.

Medical records were screened according to predefined eligibility criteria. Dogs classified as American Society of Anesthesiologists (ASA) physical status IIIE to VE undergoing emergency laparotomy for gastric dilatation-volvulus (GDV), hemoabdomen (splenectomy, hepatic lobectomy and/or bleeding masses in abdomen) were considered eligible. Dogs receiving one of the following ultrasound-guided regional techniques as part of the anesthetic protocol were included in the TPV, ESP or QL groups. Dogs managed without regional anesthesia comprised the comparison group.

Cases were excluded if local anesthetics other than ropivacaine were used, if ropivacaine concentration or dose differed from the institutional protocol, if the block was not performed according to the standardized technique routinely used by the participating anesthetists, if less than 15 min elapsed between completion of the block and skin incision, or if invasive arterial blood pressure monitoring could not be established. Cases with incomplete anesthetic records were excluded during eligibility screening.

### 2.2. Anesthetic and Perioperative Management

Following initial cardiovascular stabilization, dogs were transferred to the operating room for emergency laparotomy. Premedication consisted of intravenous methadone (0.1–0.2 mg kg^−1^; Semfortan^®^, Eurovet Animal Health BV, Bladel, The Netherlands) and dexmedetomidine (0.5–2 μg kg^−1^ IV; Dexdomitor^®^, Orion Pharma Animal Health, Espoo, Finland). Preoxygenation was provided by flow-by 100% oxygen at 100 mL^−1^ kg^−1^ min^−1^ administered for 3–5 min before anesthetic induction. General anesthesia was induced with propofol (Proposure 10 mg mL^−1^, Boehringer Ingelheim Animal Health, Lyon, France) titrated intravenously to effect and maintained with isoflurane (Isoflo, Zoetis Italia S.r.l., Milan, Italy or IsoVet Piramal critical care B.V., Barcelona, Spain) in oxygen from 50% to 100%. Standard intraoperative monitoring included electrocardiography, capnography, invasive arterial blood pressure, pulse oximetry, esophageal temperature, inspired and expired anesthetic concentrations, and end-tidal carbon dioxide. Lactated Ringer’s solution was used as the initial crystalloid fluid in all dogs. Subsequent fluid administration rates and adjustments to fluid therapy were individualized according to each patient’s clinical condition, hemodynamic status, ongoing losses, response to resuscitation, and intraoperative requirements.

Intravenous (IV) antimicrobial prophylaxis consisted of cefazolin or ampicillin (22 mg^−1^ kg^−1^) administered during the stabilization phase. No neuromuscular blocking agents were administered. Ventilatory settings were individually selected and adjusted by the attending anesthetist according to the patient’s respiratory mechanics, gas exchange, and hemodynamic status to maintain normocapnia (EtCO_2_ 35–45 mmHg); therefore, no predefined standardized initial pressure or tidal volume was applied. Maropitant (1 mg kg^−1^; Prevomax^®^, Dechra Veterinary Products A/S, Uldum, Denmark) was administered subcutaneously before transfer to the operating room.

### 2.3. Regional Anesthesia Techniques and Intraoperative Outcome Assessment

Ultrasound-guided regional blocks were performed using a high-frequency linear transducer and echogenic insulated needles selected according to patient size and operator preference. At both participating centers, ultrasound-guided regional anesthetic techniques were performed using an ultrasound system equipped with a high-frequency linear-array transducer (7–12 MHz; LA523, Esaote, Genoa, Italy). A 90 mm ultrasound-guided needle (US Simplex, B. Braun, Melsungen, Germany) was used for needle advancement under continuous ultrasound visualization.

The QL block was performed as described by Garbin et al. (2020) [9], the TPV block according to Santoro et al. (2022) [12] and the ESP block according to Cavalcanti et al. (2022) [13]. Ropivacaine 0.5% (Ropivacaina Galenica Senese, Industria Farmaceutica Galenica Senese S.r.l., Monteroni d’Arbia, SI, Italy) was administered at a total dose of 4 mg kg^−1^. For bilateral QL and ESP blocks, the calculated total volume (0.8 mL kg^−1^) was divided equally between both sides, whereas dogs assigned to the TPV group received a single unilateral injection of the entire calculated volume at the T13 level. The injection was performed on either the right or left side according to the attending anesthetist’s preference, without a predefined clinical criterion for side selection.

Correct needle placement was confirmed by visualization of hydrodissection within the intended anatomical target before injection of the local anesthetic solution. Block execution time was defined as the interval between ultrasound probe placement on the prepared skin and completion of the local anesthetic injection. Technical success was defined as ultrasonographic visualization of injectate spread within the intended anatomical plane. Block-related technical complications included failure to reach the target plane or inadvertent puncture of adjacent anatomical structures.

Presumed intraoperative nociceptive responses were defined as an increase of ≥30% in at least two of the following variables compared with the preceding 5 min interval: respiratory rate and/or patient-ventilator dyssynchrony, systolic arterial pressure and/or mean arterial pressure. Rescue analgesia consisted of intravenous fentanyl (2 μg kg^−1^; Fentadon, 50 µg mL^−1^, Eurovet Animal Health BV, Bladel, The Netherlands) administered only when the predefined criteria for presumed intraoperative nociceptive responses were met. Hypotension was defined as a mean arterial pressure < 70 mmHg and/or systolic arterial pressure < 100 mmHg.

The following variables were extracted from the anesthetic records: physiological variables, cumulative intraoperative fentanyl administration, block-related complications, intraoperative nociceptive responses and hypotension episodes. After surgery, dogs were recovered in the intensive care unit (ICU) according to each institution’s standard clinical protocols. Postoperative analgesic management was determined by the attending anesthetist in collaboration with the ICU clinicians and was therefore not standardized across institutions. Because the present study focused exclusively on intraoperative outcomes, postoperative analgesic treatments were not included in the statistical analyses.

### 2.4. Statistical Analysis

Statistical analyses were performed using GraphPad Prism (version 10.4.1; GraphPad Software, Boston, MA, USA). Continuous variables were expressed as mean ± standard deviation (SD) for normally distributed data and as median with interquartile range (IQR) for non-normally distributed data, whereas categorical variables were expressed as counts and percentages. Normality was assessed using the Shapiro–Wilk test. Between-group comparisons of continuous variables were performed using one-way analysis of variance (ANOVA) or the Kruskal–Wallis test, depending on data distribution. Categorical variables were compared using Fisher’s exact test (two-tailed). When the overall test was significant, pairwise comparisons between groups were performed, and the resulting *p*-values were adjusted for multiple testing using the Benjamini–Hochberg false discovery rate (FDR) procedure. Statistical significance was set at *p* < 0.05.

## 3. Results

### 3.1. Case Selection

**Figure 1:**
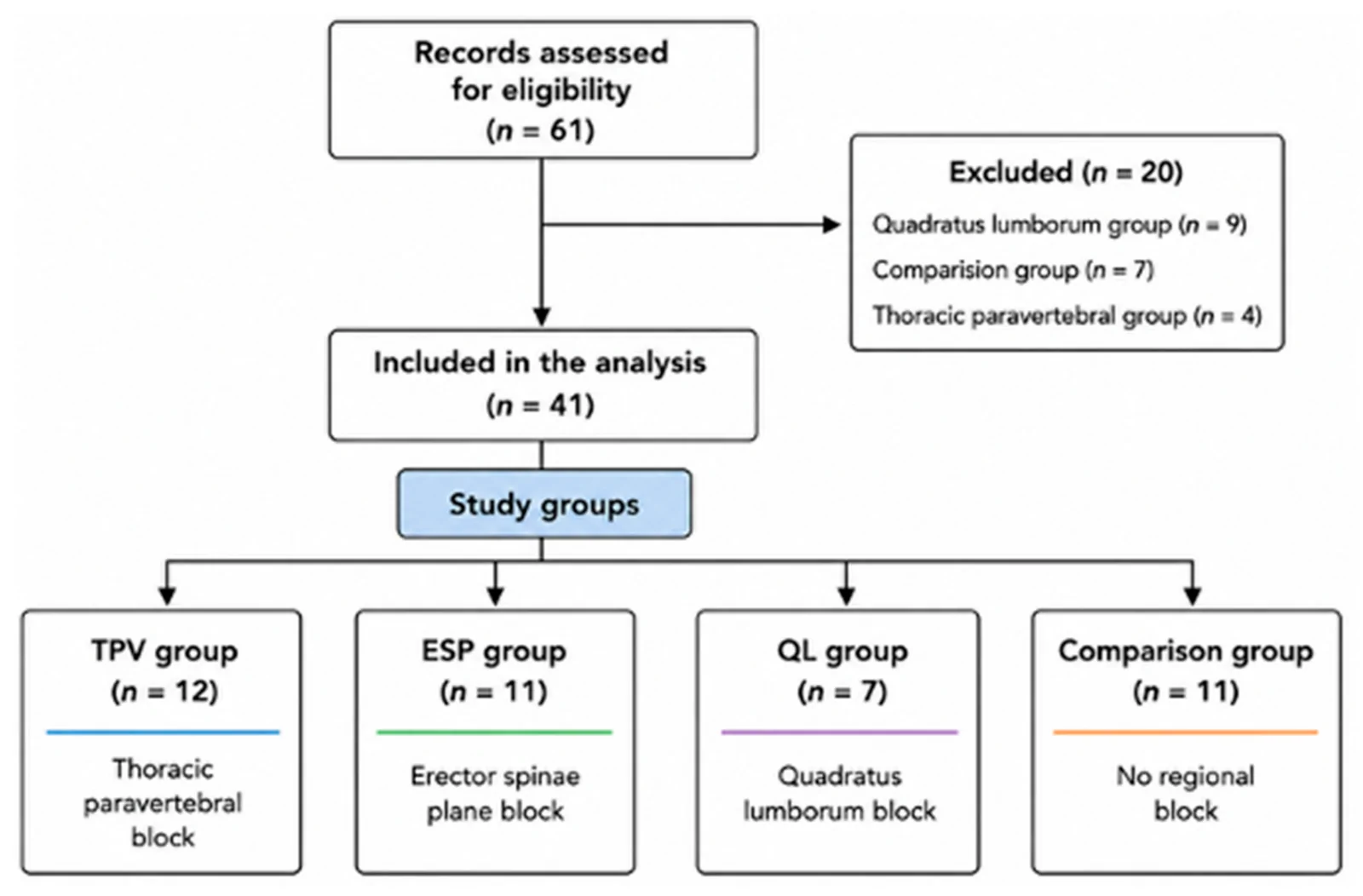
Flow diagram illustrating case selection, application of the exclusion criteria, and classification of dogs into the four study groups.

A total of 61 medical records were screened for eligibility. Following application of the predefined exclusion criteria, 20 cases were excluded, leaving 41 dogs available for analysis. The final study population comprised dogs managed with thoracic paravertebral block (TPV, n = 12), erector spinae plane block (ESP, n = 11), or quadratus lumborum block (QL, n = 7) or without regional anesthesia (n = 11). The study flow is summarized in Figure 1.

### 3.2. Animal Characteristics

In the TPV group, nine dogs underwent emergency splenectomy with concurrent gastropexy, whereas three dogs underwent emergency surgery for gastric dilatation-volvulus (GDV). Of the latter, two underwent both splenectomy and gastropexy, while one underwent splenectomy without gastropexy because of severe hemodynamic instability.

In the ESP group, eight dogs underwent emergency splenectomy with concurrent gastropexy, whereas three dogs underwent gastric repositioning and gastropexy for GDV without splenectomy, as the spleen showed no evidence of vascular compromise or other abnormalities warranting its removal.

In the QL group, five dogs underwent emergency exploratory laparotomy for hemoabdomen, including four splenectomies with concurrent gastropexy and one hepatic lobectomy. The remaining two dogs underwent gastric repositioning and gastropexy for GDV.

In the comparison group, six dogs underwent splenectomy with concurrent gastropexy, two underwent hepatic lobectomy, and three underwent emergency surgery for GDV. Of the latter, gastropexy was performed in two dogs and omitted in one because of severe hemodynamic instability.

The distribution of surgical procedures did not differ among groups (*p* = 0.91). Baseline demographic characteristics are summarized in Table 1. No significant differences among groups were observed for age (*p* = 0.67), body condition score (*p* = 0.53) or sex (*p* = 0.70). Body weight differed significantly among groups (*p* = 0.046), with post hoc analysis identifying a difference between the TPV and comparison groups (adjusted *p* = 0.05).

Mixed-breed dogs were the most frequently represented breed (13/41), followed by German Shepherd Dogs (n = 5), English Setters (n = 5), Pit Bull Terriers (n = 4), Bernese Mountain Dogs (n = 3), and Golden Retrievers (n = 3). All remaining breeds were represented by one or two dogs each.

### 3.3. Premedication, Block Execution Time and Technical Complications

All dogs received methadone and dexmedetomidine as premedication. There were no significant differences among groups in the doses of methadone (0.2 mg kg^−1^ [0.1–0.2]) or dexmedetomidine (1.5 μg kg^−1^ [0.5–2.0]) administered (*p* = 0.81 and *p* = 0.54, respectively). Block execution time differed significantly among groups (Kruskal–Wallis test, *p* < 0.0002). Median (IQR) execution times were 61 s (53–72) for TPV, 36 s (33–39) for ESP, and 53 s (46–68) for QL, with the ESP block requiring the shortest execution time. Post hoc analysis showed that TPV and ESP blocks required significantly less time to perform than QL block (TPV vs QL, *p* = 0.02 and ESP vs QL, *p* = 0.0001), whereas ESP block had the shortest execution time among the three techniques. No significant differences in predefined block-related technical complications were detected among techniques (*p* = 0.66). These included incorrect target injection, inadvertent puncture of adjacent organs or anatomical spaces, and accidental intravascular puncture.

### 3.4. Intraoperative Phase

**Figure 2:**
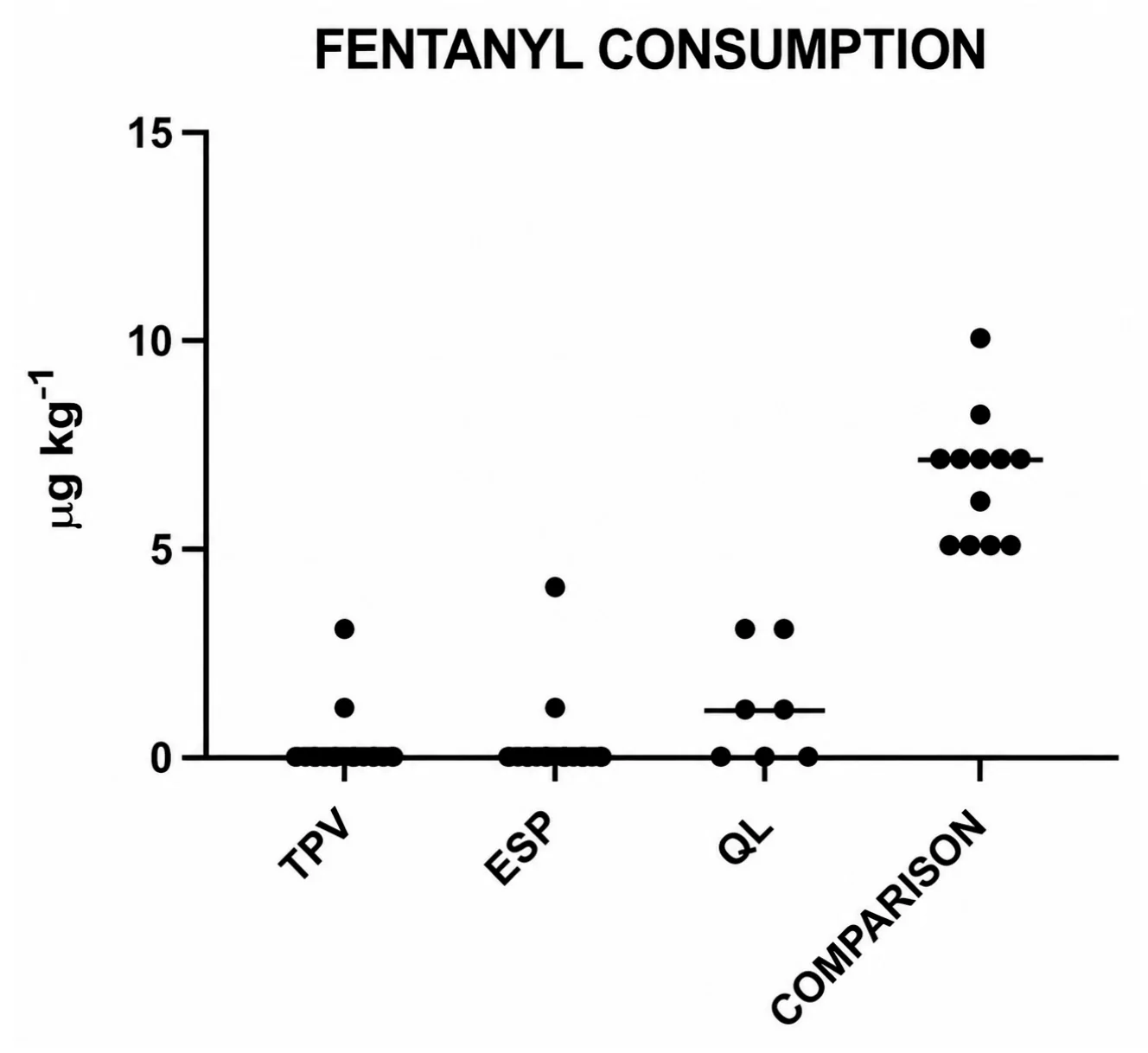
Individual total intraoperative fentanyl consumption (µg kg^−1^) according to analgesic protocol in dogs undergoing emergency laparotomy. Each dot represents one dog; horizontal lines indicate median values. TPV, thoracic paravertebral block; ESP, erector spinae plane block; QL, quadratus lumborum block; Comparison, systemic analgesia without a regional block.

Regarding intraoperative analgesic requirements, total fentanyl consumption differed significantly among groups (*p* < 0.0001). Post hoc pairwise comparisons showed that fentanyl consumption was significantly lower in the TPV, ESP and QL groups than in the comparison group (adjusted *p* < 0.0001 for all comparisons). Median (IQR) intraoperative fentanyl consumption was lowest in the TPV group (2 µg kg^−1^ [0–2.5]), followed by the ESP (3 µg kg^−1^ [0–4]), QL (5 µg kg^−1^ [2.5–6]), and comparison groups (10 µg kg^−1^ [4.5–12]). Rescue fentanyl was required in 2/12 dogs in the TPV group, 2/11 dogs in the ESP group, 4/7 dogs in the QL group, and all dogs in the comparison group (11/11) (Figure 2).

At least one episode of intraoperative hypotension occurred in 21 of 41 dogs (51.2%). The incidence differed significantly among groups (*p* = 0.02). Post hoc analysis demonstrated a significantly lower incidence of hypotension in the TPV and ESP groups compared with the comparison group (adjusted *p* < 0.001 and *p* < 0.0001, respectively), whereas no significant difference was observed between the QL and comparison groups.

## 4. Discussion

The principal finding of this multicenter retrospective study was that ultra-sound-guided thoracic paravertebral (TPV), erector spinae plane (ESP), and quadratus lumborum (QL) blocks were associated with lower intraoperative fentanyl requirements in dogs undergoing emergency laparotomy. In addition, TPV and ESP, but not QL, were associated with a lower incidence of intraoperative hypotension compared with systemic analgesia alone.

These findings were obtained in dogs undergoing emergency surgery for gastric dilatation-volvulus or hemoabdomen, conditions frequently associated with hypovolemia, hemorrhage, impaired tissue perfusion, systemic inflammation, and reduced cardiovascular reserve [1,2,14,15,16]. Block-related technical complications were uncommon, and ESP required the shortest execution time. However, because the regional technique was selected according to clinician preference rather than random allocation, the observed differences should be interpreted as associations rather than evidence of a causal analgesic or hemodynamic benefit.

Current evidence supporting TPV, ESP, and QL blocks is largely derived from elective procedures. Previous studies have demonstrated reduced intraoperative opioid requirements or satisfactory perioperative analgesia following QL block in dogs undergoing orchiectomy, ovariohysterectomy, and laparoscopic ovariectomy [10,11,17,18]. Likewise, caudal TPV improved perioperative analgesia during radical mastectomy, whereas ESP reduced intraoperative nociceptive responses during elective ovariohysterectomy [12,19]. TAP techniques have also demonstrated opioid-sparing effects in laparoscopic ovariectomy and mastectomy [19,20,21]. More recently, Fontana et al. (2026) reported lower fentanyl consumption and inhalant anesthetic requirements following bilateral QL block in dogs undergoing elective and emergency abdominal surgery [22]. Unlike that study, which evaluated QL block alone in a heterogeneous surgical population, the present study focused exclusively on emergency laparotomy while directly comparing three regional techniques with systemic analgesia alone. The reduced fentanyl requirements observed in all regional anesthesia groups are therefore consistent with previous reports [10,11,12,18,19,20,21,22].

Nevertheless, cumulative fentanyl administration should be regarded as a pragmatic surrogate of rescue analgesic requirement rather than a direct measure of nociception. Rescue fentanyl was administered according to predefined cardiovascular and respiratory criteria, which may also have been influenced by anesthetic depth, mechanical ventilation, hemorrhage, fluid therapy, vasoactive support, pre-existing cardiovascular dysfunction, and concomitant anesthetic drugs [3,16,17,23,24,25,26,27]. Consequently, the present findings support an opioid-sparing association but do not independently demonstrate a more complete somatic or visceral blockade.

Interestingly, TPV and ESP, but not QL, were associated with a lower incidence of intraoperative hypotension than systemic analgesia alone. This partially contrasts with the retrospective study by Fontana et al. (2026), in which QL block reduced fentanyl and inhalant anesthetic requirements without affecting hypotension or the need for cardiovascular interventions [22]. Although reduced opioid or inhalant administration may have contributed to the observed hemodynamic differences, arterial pressure during emergency anesthesia is influenced by numerous factors, including disease severity, hemorrhage, circulating blood volume, resuscitation status, inhalant concentration, fluid and blood-product administration, vasoactive support, and concurrent anesthetic drugs [3,16,17,23,24,26,28]. As these variables were not controlled in the present retrospective analysis, the lower incidence of hypotension observed in the TPV and ESP groups should be interpreted cautiously and cannot be attributed solely to the regional techniques or to reduced fentanyl administration. Maropitant should also be considered among the potential pharmacological contributors to the observed hemodynamic response. Intravenous administration has been associated with transient decreases in arterial blood pressure, although recent evidence suggests that clinically relevant hypotension may be less pronounced in dogs premedicated with dexmedetomidine [29]. In the present population, maropitant was administered subcutaneously and dexmedetomidine was included in the premedication protocol, which may have limited the clinical relevance of this effect, although this cannot be confirmed from the present data. Moreover, recently reported antinociceptive effects of maropitant based on PTA index assessment [30] should also be acknowledged as a potential contributor to the perioperative analgesic response.

The three regional techniques investigated differ substantially in their anatomical targets and proposed mechanisms of action. TPV deposits local anesthetic adjacent to the thoracic paravertebral space, whereas ESP and QL are interfascial plane blocks whose clinical effects depend on injectate spread toward neural structures involved in somatic and visceral nociception [5,6,7,8,9,10,11,12,13]. Although cadaveric studies have demonstrated potentially relevant injectate distribution, anatomical spread does not necessarily predict clinical analgesia [7,8,9,13]. Consequently, the present study cannot determine whether the reduced fentanyl requirements resulted from somatic blockade, visceral analgesia, reduced inhalant requirements, or a combination of these mechanisms.

Likewise, the shorter execution time observed for ESP should be interpreted cautiously, as procedure duration was likely influenced by differences in technique, operator experience, and clinical workflow [5,9,13]. Nevertheless, shorter execution time may represent a practical advantage when rapid surgical intervention is required [1,2,23].

Regional anesthesia should be considered part of a multimodal analgesic strategy rather than a replacement for systemic analgesia. TAP block remains a valuable option for abdominal wall analgesia but provides limited visceral analgesia [5,6,20,21]. Similarly, TPV, ESP, and QL should be integrated with systemic analgesic protocols in dogs undergoing emergency laparotomy [5,31,32]. Neuraxial anesthesia represents another potential alternative. Although epidural blockade provides effective segmental analgesia, its cranial spread is influenced by several technical and anatomical factors and may increase sympathetic blockade, hypotension, and respiratory compromise, particularly in hypovolemic or hemodynamically unstable patients [5,23,33]. In elective ovariohysterectomy, epidural and QL blocks have provided comparable analgesia and cardiorespiratory stability [34]. However, these findings cannot be directly extrapolated to dogs undergoing emergency laparotomy with hemorrhage, GDV, or systemic inflammation. The low incidence of block-related complications supports the technical feasibility of these techniques when performed by experienced anesthetists under ultrasound guidance. However, the study was not powered to detect uncommon adverse events, and block success was inferred from ultrasonographic visualization of injectate spread rather than objective sensory assessment. Therefore, these findings should be interpreted as supporting technical feasibility rather than establishing the overall safety profile of the techniques [5,7,8,9,13].

This study has several limitations inherent to its retrospective design. Anesthetic depth, fluid therapy, vasoactive support, ventilation, and rescue analgesic administration were not standardized and may have influenced intraoperative fentanyl requirements independently of block efficacy. Selection of the regional technique also reflected clinician preference, introducing selection bias and potential confounding by indication. In addition, the inclusion of different emergency conditions and surgical procedures introduced unavoidable variability in nociceptive stimulation, blood loss, cardiovascular impairment, and resuscitation requirements [1,2,14,15,16,17,23,24]. Finally, postoperative analgesia was not standardized; therefore, the persistence of the intraoperative opioid-sparing effect and its influence on recovery, postoperative pain, complications, hospitalization, or survival could not be assessed [17,23,32].

## 5. Conclusions

Ultrasound-guided TPV, ESP, and QL blocks were associated with lower intraoperative fentanyl requirements in dogs undergoing emergency laparotomy. TPV and ESP were also associated with a lower incidence of intraoperative hypotension than systemic analgesia alone, while block-related complications were uncommon. These findings support the use of ultrasound-guided truncal regional anesthesia as part of a multimodal anesthetic strategy in selected emergency abdominal patients. Prospective controlled studies are warranted to compare the efficacy of individual techniques and determine whether intraoperative opioid sparing translates into improved perioperative outcomes.

## Figures and Tables

**Table 1:** Baseline characteristics of the study population. Data are presented as median (IQR) or counts. * Post hoc comparison: TPV vs comparison group, *p* = 0.05.

Variable	TPV	ESP	QL	Comparison	*p* Value
Age (years)	9 (8.25–10)	9.5 (8–11)	9 (6–10)	10 (9–13)	0.67
Body weight (kg)	38 (27–40)	23 (18–33)	30 (25–43)	22 (19–28)	0.046 *
Body condition score (1–9)	4 (4–5)	4 (4–5)	4 (4–6)	5 (4–6)	0.53
Sex (M/F)	5/7	7/4	4/3	6/5	0.70

## Data Availability

The original contributions presented in this study are included in the article. Further inquiries can be directed to the corresponding authors.
